# Book Reviews

**Published:** 1832-06

**Authors:** 


### CRITICAL ANALYSES.

**Quæ laudanda forent, et quæ culpanda, vicissim,**

**Illa prius, cretâ; mox hæc, carbone, *notamus.*—Persius.**

*The Anatomy of the Thymus Gland.* By Sir Astley Cooper, Bart., F.R.S., Sergeant Surgeon to the King, Consulting Surgeon to Guy's Hospital, &c.—4to. pp. 47; with Plates. Longman, London.

Although every writer on the anatomy and physiology of the human body gives some description of the structure of the thymus gland, and many offer their speculations as to the functions nature has designed it to perform, it is certain that but very few have themselves examined this curious and perplexing organ; the profession will therefore be glad to receive from such distinguished authority an account of the anatomy and physiology of this gland, derived not from the observations of others, but from dissections performed by the hands of Sir Astley Cooper.

In a brief preface Sir Astley states, with his characteristic urbanity and anxiety for the diffusion of knowledge, that, as the preparations which form the foundation of these observations on the structure of the thymus gland are carefully preserved, it will at all times afford him great pleasure to exhibit them to those of his professional brethren, whether domestic or foreign, who are zealous in the science of anatomy.

In the human subject, the thymus gland is difficult of investigation, from the smallness of its size, and the extreme delicacy of its texture. Sir Astley confesses it would have been scarcely possible to learn the most important parts of the structure of this gland, by the examination of it, in the human subject alone. From being aware of its great magnitude in the fœtal calf, he commenced his inquiries in that animal and the lamb; and it was found that the difficulties of the investigation were greatly diminished; "for I was able to inject it, dissect all its parts, shew their relative situation, learn the structure by which the fluid is secreted, the cavities containing it, the vessels by which the fluid is carried away, and to collect it in sufficient quantity to make it the subject of chemical analysis." We doubt not there are many in our profession, as well as in all other pursuits, who approach the investigation of every subject with apathetic indifference that does not promise to return some immediate practical advantage: the more scientific members of it, however, will agree with Sir Astley "that every portion of the animal body, however minute, should be carefully traced, and accurately known;" and that the thymus gland should not be neglected must be evident, as "when the size of the organ is considered; when the quantity of fluid it secretes, both in the human body and quadrupeds, is recollected; when the important situation it occupies near the heart, and upon some of the largest vessels of the body, is remembered; as well as its appearing at the fœtal and infantile period only, it cannot be doubted but that the function which it performs is highly essential to the existence and growth of the fœtus and infant."

Sir Astley gives a detailed and very interesting account of the general form and organization of the thymus gland in the fœtal calf, from which we find that each lobe is made up of numerous small secreting cells, and of larger cavities or reservoirs; and each of the larger lobes is connected by a tube surrounded by a portion of glandular substance. Thus the organ is constituted of lobes, having secreting cavities on the outside, reservoirs within, and a vessel of communication from lobe to lobe. After having described the blood-vessels of the organ, Sir Astley traces the absorbent vessels and their glands.

"These vessels are formed to convey the fluid of the thymus gland into the veins, although their size is so large as readily to admit of their being injected with wax, yet I believe them to be more of the structure of absorbent vessels than of excretory ducts.

"An excretory duct is in itself a gland (for example, the ureter); it is generally a muscular tube on the outer side and a secreting membrane within; is free from any valvular apparatus excepting at its termination as the ureter and common duct of the liver. But the vessels I am now describing, although of large size, are transparent and possess valves, and, above all, if quicksilver be thrown into the absorbent glands of the thymus, small vessels are filled from them which open suddenly into a tube of considerable diameter, forming the two vessels I have mentioned; and further, to shew that they partake more of the nature of absorbent vessels than the structure of an excretory duct, they cannot be injected but in their course towards the veins from the valves which they contain.

"Around the thoracic portion numerous absorbent glands are found which send vessels into the veins at the junction of the jugulars with the superior cava.

"These vessels I consider and shall name absorbent ducts of the gland, and they are the carriers of the fluid (hereafter to be described,) from the thymus into the veins of the lower part of the neck." (P. 14.)

The comparative anatomy of the gland in the dog, kitten, ass, lamb, and pig, is briefly described; but, upon the whole, the thymus of the calf and lamb are more readily investigated, and made into preparations, than those of any other animals that the author has examined.

The structure and relative situation of the gland in the human subject are next pointed out, and a particular account is given of a fascia interposed between the thymus, the curvature of the aorta, and the great arteries which arise from it, and also the trachea. From repeated dissection of the thymus in the human subject, the following account is given by Sir Astley:

"1st. It is composed of a gland on each side, united only by cellular membrane.

"2d. It is formed of two ropes which can be with care unravelled, and they are of considerable length.

"3d. The ropes are constituted of small and large lobes which appear as knots upon the rope.

"4th. These are disposed in a spiral or serpentine course, from the upper part of the cervical, to the lower extremity of the thoracic portion.

"5th. Each portion of the rope is a secretory structure.

"6th. The lobes contain secretory cavities or cells, which may be readily shewn by filling the gland with alcohol, air, gelatin, or even wax.

"7th. A pouch of communication exists between the lobes and the reservoir.

"8th. The gland has a central cavity or reservoir.

"9th. This cavity is not straight, but spiral or serpentine.

"10th. The reservoir is lined by a very vascular mucous membrane.

"11th. The ropes of the gland pass in a spiral or serpentine direction around the mucous membrane, which line and principally forms the reservoir, and these ropes, being united by that membrane to each other, assist in forming the cavity.

"With respect to the arteries of this organ, they are principally derived from two sources. Each thoracic portion is supplied by a branch which is sent off by the internal mammary. It enters at the junction of the cervical with the thoracic part, generally on their outer side, but sometimes between the cervical portions, and descending upon the middle of the gland, divides to supply the spirally disposed lobes.

"The vessel passes to the inner side of the reservoir and is distributed to its mucous membrane on the one hand, and to the glandular structure on the other.

"The other principal artery of the thymus is sometimes derived from the superior thyroideal, at others, from the inferior thyroideal, artery, and, descending upon the lobes of the cervical portion, passes into them, and to the membrane of the cavity which they contain, and, ultimately, anastomoses with the branch from the mammary artery. These arteries, besides supplying the gland with blood, serve the purpose of combining their lobes and preventing their separation; for, until they are divided, the ropes cannot be unravelled.

"The venæ thymicæ have a different course to the arteries; for, although the internal mammary and thyroideal veins receive small branches from this gland, yet the principal veins are those which end in the vena innominata.

"A considerable vein springs from each thoracic portion, and it passes from the posterior surface of the thymus into the vena innominata; having received a branch from the cervical portion, and vessels from the thoracic, it is found near the centre of the gland.

"A very small vein enters the thyroideal from the cervical portion, and this vein anastomoses with that of the thoracic part.

"The absorbent vessels I have only once been able to inject in the human subject proceeding from an absorbent gland of the thymus.

"Absorbent glands are found at the upper part of the sternum in the mediastinum; also a small gland, between the thoracic portions, and some at the junction of the thymus with the jugular and subclavian veins, where the principal trunks of the absorbent vessels at all periods of life terminate.

"Here the advantage of comparative anatomy is evinced, in the readiness with which the absorbent vessels, their glands, and the absorbent ducts, can be shewn in the fœtal calf." (P. 32.)

The nerves of the thymus are very minute.

*Physiology of the Thymus Gland.* Sir Astley differs from the opinion of Hewson.

"I cannot agree with the opinion that the structure of the thymus and absorbent glands is similar: one is conglobate, and the other conglomerate; one is firm and compact, and the other is loose and pulpy; the one contains cells of considerable magnitude when in a distended state, whilst in the absorbent glands the cavities are small and with so much difficulty traced that there is still a doubt if they be cellular or vascular.

"The office which the thymus is designed to perform is evidently connected with the fœtal stages of existence, as it gradually lessens soon after the child is born, and, even when the gland remains of considerable bulk, its secretory cavities are much diminished." (P. 38.)

An accurate analysis is given, obtained by the author from Dr. Dowler, of Richmond, of the fluid contained in the thymus.

Sir Astley considers it erroneous to suppose that this gland continues for some time after birth to perform the same office as that which it supported in the fœtal state. In a child of one month old, the lobes had become quite thinned by absorption, and one of the reservoirs was partially obstructed. In a child of four months, the gland weighed about five times less than in the fœtal state. In a calf of four months, the gland is very large, yet the cells and reservoirs will not receive one half of the injection which will enter them in the fœtus of nine months. Sir Astley, therefore, puts the following ingenious query:

"As the thymus secretes all the parts of the blood, viz. albumen, fibrin, and particles, is it not probable that the gland is designed to prepare a fluid, well fitted for the fœtal growth and nourishment, from the blood of the mother before the birth of the fœtus, and consequently before chyle is formed from food, and this process continues for a short time after birth, the quantity of fluid secreted from the thymus gradually declining as that of chylification becomes perfectly established?" (P. 44.)

*Disease of the Gland.* Parts which have ceased to perform their functions, as the mamma, after menstruation and parturition, so frequently degenerate into diseased changes, that morbid affections of the thymus might be expected to be frequent; yet, in the course of forty years' experience, Sir Astley Cooper has only witnessed the following single example of it. Varieties in the size of the gland are of common occurrence, but disease of its structure is extremely rare.

"I was requested to visit a young person, nineteen years of age, who suffered under so severe a dyspnœa, that it was with great difficulty she could remain recumbent for a few minutes, and, if a short period of repose was obtained, she started up with a sense of suffocation, and for several seconds struggled for breath.

"Upon inquiring into the cause of her suffering, I found a swelling which occupied the inferior part of the neck at the upper opening of the thorax, which projected above the clavicle upon each side, and as I supposed arose from an enlargement of the absorbent glands at the termination of the jugular and subclavian veins.

"The swelling had existed for many years, but of late suddenly increased. I ordered leeches to be applied, her bowels to be opened, and on the following day she was somewhat better, but another day brought with it not only her former, but still more aggravated, sufferings; I then advised a blister to the upper part of the sternum and to the swelling in the neck; desired the cuticle to be removed, the part to be dressed with the unguentum hydrargyri, and directed her to take calomel and opium, which she accomplished without much difficulty, as her deglutition was less affected than her breathing.

"The means which I recommended gave her only slight temporary relief, and she became daily weaker; her legs were œdematous, and she was unable to get any rest, but in the sitting posture, and then only with her head inclined forward, and supported in that position by her sisters; for the moment it fell back, the pressure of the tumor on the trachea and the dyspnœa were suddenly increased.

"I witnessed her making daily approaches to dissolution, without being able to afford her any permanent benefit; she died after a fortnight, not from any sudden attack of suffocation, but from being worn out by the constant irritation excited by the difficulty in respiration.

"I obtained permission to examine the body, and found that the disease was situated in the thymus gland; the swelling reached from the curvature of the aorta to the lower part of the thyroid gland, and the latter was also considerably enlarged.

"The thymus appeared of a yellowish white colour, and was divided into several large lobes.

"The trachea was involved in the tumor, and its sides were compressed by it, so that its transverse diameter was somewhat diminished. The arteria innominata was placed behind it, and the left subclavian, and left carotid arteries to its left side; it surrounded the vena innominata, and, upon cutting into the vein, the diseased gland was found projecting into its cavity; and, upon making an incision into the swelling, the reticular texture of the gland was found to be filled by a white pulpy substance.

"In this case, the complaint was compounded of a diseased growth of the thymus and of bronchocele, or an unnatural growth of the thyroid gland. The latter is so placed, that its enlargements little endanger suffocation, because the surrounding parts can yield to the pressure of the swollen gland; but, as the thymus is situated in the thoracic opening, in its enlarged state it soon reaches the sternum and first rib, by which it is bound, and, therefore, its increase is towards the trachea, which becomes enveloped by it, and its function interrupted in consequence of its compression.

"The disease appeared to be of the fungoid kind." (P. 44.)

The plates, by CHILDE, at the termination of the work, are admirably executed, and convey a very clear idea of the structure of the thymus gland, as it is exhibited by the elaborate dissections of Sir Astley Cooper.

In conclusion we must observe, that the anatomical description which Sir Astley gives of the thymus gland is perfectly original, and that its accuracy is most satisfactorily established by a splendid series of preparations which he has made, for the purpose of illustrating its structure from the earliest periods of fœtal life to the time of its disappearance after birth, both in animals and the human subject. In viewing these preparations with Sir Astley Cooper, we felt a double gratification: we were much instructed by his description of them, and we could not witness the great enthusiasm and patience with which he was content to go over a subject that to him must have lost the charm of novelty, merely for the edification and improvement of others, without feeling increased admiration at the unwearied industry of a man who might long ago have ceased his labours, and yet have been certain of the highest degree of professional renown.

---

*Dissertations on Malaria, Contagion, and Cholera; explaining the Principles which regulate Endemic, Epidemic, and Contagious, Diseases, with a View to their Prevention: intended as a Guide to Magistrates, Clergymen, and Heads of Families.* By WM. AITON, M.D. Member of the Royal College of Surgeons of London; Extraordinary Member of the Royal Medical Society of Edinburgh; and Surgeon of the Royal Navy.—8vo. pp. 308. Longman and Co , London.

AT a time like the present, when the doctrine of contagion has been so vehemently discussed, when debates on this one point alone have been protracted beyond precedent, when scarcely any other subject has been mooted in some of our medical societies during an entire session; when, seeing how little their elders were agreed, every tyro has presumed to deluge the public ear with his opinions upon a topic, concerning which he knew practically nothing; when the several parties who have waged for so many months a wordy war, seem to the by-standers to have been often contending rather for the palm of victory than for the cause of truth; a publication such as this which Dr. Aiton has given to the world cannot but be considered as exceedingly opportune in the epoch of its appearance, and well calculated to achieve an important good: for here, within a very convenient compass, will be found a luminous statement not only of the philosophic author's views, based on much original observation, and supported by the coincident observations of contemporaries, but also a very satisfactory digest of the recorded opinions of the most celebrated physicians and surgeons of former ages; to which, perhaps, they who are accustomed to generalise upon solitary cases will be persuaded to pay more respect than they have hitherto done either to verbal or written authority alone.

To this volume, therefore, we could be well content to refer those who, on the one hand, (as we have heard them with our own proper ears,) assert that all diseases are contagious, more or less; and, on the other, that none, not even variola, is communicable from man to man:* for here they would learn enough to make them more guarded in their asseverations, and careful in their language; for it is evident that they who argue thus apply to words in common use uncommon significations; a plan admirably calculated to mislead their hearers and confound themselves, and good for nothing else.

Whether the words contagious and non-contagious are the best possible terms that could have been devised to express two very different ideas, is a point of little comparative importance: suffice it to know, that the ideas they express are different, and that they have long been used to signify, the one a disease which *is*, and the other a disease which is *not*, communicable from man to man.

Diseases which the majority of medical philosophers admit to be contagious, are not all equally, nor at all times, communicable. This is a point which hasty disputants too generally overlook; and another, which they too frequently neglect, is that extraneous circumstances are not all equally, nor at all times, favorable to their communication. The propagation of some diseases is encouraged by cold, others by heat; some attack the under, others the over, fed; and some are no respecters of persons or of seasons: and yet, notwithstanding the notoriety of this, the negative fact that certain persons have escaped contamination, although within the sphere of a contagious disorder, has been presumed to out-

* "Nihil est tam absurdum," &c. Some have even denied that syphilis *ever existed*: a book having this object was some years since published at Strasburg, and we think the author had reason quite as much on his side as those who deny that it is contagious.

weigh the positive evidence that others, previously in a healthy state, have, under similar circumstances, contracted the disease. Again, although it is continually confessed that diseases of non-contagious origin (as typhus) may become contagious, and be communicated from man to man, still this admission is too often a barren knowledge, and they who make it go on arguing as if it had never been made.

If what we have said be true of diseases in general, it is much more strictly so when applied to that plague which has lately come among us; when applied to that form of cholera which, arising in the Delta of the Ganges, has spread from India through Persia to Russia, and having slightly afflicted our land in comparison to the havoc it has committed elsewhere, now rages with fearful violence in the capital of France: for cholera is declared by many to be non-contagious, because persons *may be* exposed to its influence without being affected, although others who are similarly exposed take the disease and die.

But these are topics which have all been well discussed by our author, and we only regret that our space will compel us to make much fewer extracts from his work than we could wish; for in the volume before us the subject is treated on a broad and philosophic basis, and, while cholera forms a prominent feature, it is not allowed to engross an undue proportion of these pages; and probably by this plan more light is thrown upon its peculiarities than when, as is often the case, an over-preponderating prejudice bends and warps the facts to suit a theory which should rather have been made to accommodate itself to them.

The two dissertations now published, the one on "Malaria," the other on "Contagion," are said to form only part of a larger work "on the Principles of Health;" but these are printed separately, as the author thinks they may be useful to the public in the present crisis; and, if the public will reflect upon the matter they contain, we think so too. He observes in the preface,

"Those who have not had opportunities of making themselves acquainted with the history of epidemic diseases, may blame me for laying too little stress on the influence of the atmosphere; but, when it is considered how often mankind have been misled by wrong theories concerning it, and how little statistical medicine has been regarded by many even of our best authors on epidemic diseases, I hope it will be conceded that I have given to both doctrines a full and fair examination.

"There is scarcely a great epidemic, plague, typhus, or yellow fever, on record, which was not attributed, at its commencement, chiefly to some known or hidden quality of the atmosphere, while the doctrine of contagion and importation was rejected as a vulgar belief, for the want of what is called direct evidence; every wind that prevailed, and every accidental change of weather which happened at the time, were not only eagerly laid hold of by those who believed they knew more about the matter than their fellow-mortals, but were at once held forth by them as the most convincing evidence. The fatal consequences of such vague and conjectural notions were just what might have been expected. Before the real causes of the evil became manifest to all unprejudiced persons, the diseases in most instances gained such ground, that all human efforts to stop their progress proved unavailing, and thus the prejudices of the ignorant as to the efficacy of human means of prevention were sure of being strengthened.

"The injurious tendency of wrong notions regarding the general atmosphere has not been confined to great epidemics. This heart-reviving fluid has been blamed for many of the evils occurring in private life: thus, the fashionable lady who injures her health and her looks by late hours and crowded assemblies, the pampered profligate who derives his illness from his own dissipation, the bon-vivant who indulges himself into gout, the delicate female starved into a consumption by improper clothing and food, with many more unhappy and unfortunate invalids, have all recourse to the general atmosphere, as affording an easy and ready solution of every pathological difficulty.

"Let me, therefore, appeal to the good sense of the public on the subject of cholera, and let me conjure them not to throw away the lessons afforded by past experience. Those who peruse the flimsy productions of the newspaper press on the subject of its atmospheric origin, will not blame me for over-zeal, if they calmly consider the principles on which such theories are founded. 1st. We are told that undue proportions of the gases composing the atmosphere are the causes of cholera, though the same quantity of each be found in the air of different countries and at different heights from the earth's surface. 2d. Cholera depends upon certain winds; yet it has often been confined for a time to a single spot of the countries it visited. When it does break out, it does not spread like influenza over a whole community, but is confined at first to one individual, to one room, to one house, to one street, to one town; it then goes to another place to observe a similar mode of procedure. In shifting its residence, it progresses with equal facility contrary to the wind as it does with it, and it continues its ravages in a place during every kind of wind as when there is no wind. 3d. It arises from climate and season, although it breaks out in different places at uncertain periods, and in all climates and seasons alike. Vast tracts of the inhabited parts of the globe have not yet been visited, although they possess the climate and seasons supposed to be favorable to its existence. 4th. Cholera derives its origin from certain soils; yet marshy, sandy, and clay, soils, in every condition of moisture and dryness, with shingle, rich loam, poor gravel, and hard rock, produce it. In other words, places possessing every kind of soil and places having no soil. 5th. It owes its origin to vegetables; yet it rages in ships at sea, where men die from the want of vegetables. 6th. Certain localities produce it, although it marches with seeming indifference in cultivated and in barren places, over mountainous districts the same as on level plains, over water as on dry land, and a great proportion of the civilized world has fallen under its malign influence. Lastly, Cholera is guided by the electric fluid; yet its rapidity in travelling, never exceeds that of human intercourse in the countries it visits. Such is a synopsis of the atmospheric theory of cholera. To give credence to such a theory, is to believe in the existence of something arising from nothing, yet a being independent of everything. On the other hand, if cholera be compared with other contagious epidemics, an account of which I have given in the following pages, the reader will be the better able to form a correct notion regarding its nature and origin." (*Pref.* p. iii.)

At almost every other epoch we should have made our selections chiefly from the first dissertation, which contains many important remarks on medical geography and topography; but as it behoves us to be obedient to the signs of the times, we shall pass by these, that we may devote more space to the consideration of contagious epidemics, and especially of Asiatic cholera.

Much has been said of the irregular journeyings of diseases considered as contagious, of their occurrence at different extremities of large towns, and their passing over many intermediate places, as well as of the greater liability of the poor to be attacked, and the comparative safety of the well-clothed and fed; a too pertinacious dwelling upon which we fear has often closed the half-opened hand of cold-hearted charity. Let them, however, who will not give out of love, give out of fear; for they may rest assured, that to relieve others is the most efficient means to defend themselves. On both these subjects our author reasons well, and he concludes by observing, "Need we wonder or complain if Providence has decreed that the diseases which arise thus among the poor should recoil upon the rich, as a just punishment for their want of charity?"

"Epidemics have been sometimes observed to break out amongst relations, living in different quarters of the town, at, or near the same time. Those who have witnessed the conduct of strangers, on their first arrival in a place, will not be surprised at this, even if the persons so taken ill had not been related. The aerial theorists, however, explain it on the principle of similarity of constitutions observed or supposed to exist amongst persons nearly related by blood to each other; they leave out altogether the greater probability of intercourse between such relations. We shall suppose a thing by no means unlikely, that one of the strangers, prompted by the ties of relationship, friendship, or from interest, called, on his arrival in port, at the house of an individual; he is almost certain, in either of these cases, to be carried straight to the houses of some of the relations of the individual on whom he called. May not a person convey contagion to others, though in perfect health at the time himself? May not the goods he sells or exchanges, or the presents he gives away, do the same thing? Intercourse in a large town does not always depend on proximity: very frequently, next neighbours do not exchange visits for years; sometimes they do not know each other's names or occupations. Occasionally epidemic diseases are irregular and whimsical in their mode of procedure; they often spread for a time in one direction chiefly, then suddenly change their course to another. At one time they travel with the wind, but in an opposite direction soon after. They frequently prefer one side of a street, or a particular quarter of a town. Sometimes they do not appear in the town for a time, but make nearly a circle round it. Having performed this office in the suburbs or outskirts, they suddenly take a turn to the country by a favorite route, then return directly to the centre of the town, which is now infected for the first time during the rage of that epidemic. In what way can this be explained, but by referring to that whimsical and capricious being, man, himself.

"Smugglers, thieves, vagabonds, and vagrants of all descriptions, have been carriers and importers of contagion in every country. Few of those persons are regulated by law or rule of any kind. Beggars are said to travel with the wind generally; pedlars will not be fond of travelling against it; gypsies have never been remarkable for the regularity of their procedure. As smugglers, thieves, and vagrants, have reasons for keeping out of the view of the police, many of them being banished from the towns, they take up their abodes in the suburbs or outskirts, where they are beyond the bounds of police, and where the want of lamps is favorable to their views. Suburbs are chiefly inhabited by two very opposite classes of people; persons from the country, and persons from the town. The former are attracted to towns to spend their money, educate their children, or put them to trades and professions. They often prefer the suburbs on account of the air being purer, house-rents cheaper, exemption from town-burdens, and being, as they express it, both in town and country. Besides, they believe their children run a less risk of temptation from vice or bad example. A considerable proportion of those from town, on the other hand, are persons, who, as the phrase is, have outrun the constable in different shapes: too well known in every quarter of the town, they remove to obscure parts of the suburbs, amongst strangers to whom they are not known. Many of these persons prowl about the outskirts in every direction during the night, where, favored by the darkness and absence of police, they commit all kinds of depredations.

"Suppose, then, a criminal, just liberated from jail, where his clothes have become impregnated with the seeds of disease, is banished the city, and goes to haunt with his numerous friends in the suburbs, who could answer for the regularity of his actions, or tell what rout he will take? Again, a beggar woman, who lives in the suburbs, borrows a child ill of small-pox, for the purpose of exciting charity: she cannot beg in town, but proceeds in a circular course round it; and, after going this round, she travels directly to the first village in the country. The disease, in this case, would observe the tract of the woman; first round the town, then to the village in the country by the same route, and, once spread in the village, it would be readily brought back to town by the villagers, who, not being, like the beggar, proscribed the bounds of police, would carry it to its centre.

"The greater partiality of epidemic diseases to one side of a street depends upon difference of intercourse and communication. In most great commercial towns a marked difference may be observed between one side of a principal street and another. Such is the difference in this respect, that, in innumerable instances, the shops of one side let for double the rent of those of the other, even although the accommodation should be similar.

"Such is our plain explanation of the occasional whimsical procedure of epidemic diseases. Those who imagine that a contagious disease should, like a volcano, vomit out its flame in all points of the compass alike, might as well compare a common conflagration to a volcano, and expect that the small spark of fire which kindles one should spread in all directions alike, without reference to the fuel which supplies it. A contagion, like small-pox, often spreads rapidly in the villages in Britain. The inhabitants are not so much crowded together as the people in large towns, and the circulation of air is freer; but these advantages are more than counterbalanced by the habit of family visiting being more general. Every individual in a village is known to every other individual. No sooner is a person taken ill of a dangerous disease, than the news becomes known from one end to the other, and all the friends and gossips of the place pay their visits of ceremony or condolence.

"If a contagion happens to be less portable or more volatile, it will not spread in villages so much as in towns, particularly in a hot climate. Where, however, the houses of villages are as much crowded together as those in towns, as is the case in Barbary, then the disease will spread rapidly. Diseases arising from malaria will likewise be observed sometimes to prevail more on one side of a street; a circumstance as easily explained as that the one side should have the sun in the morning, the other in the afternoon. The stone of the houses built on one side, though of the same age and same material, nay, even built by the same individual, has a different colour from difference of exposure. In like manner, the north side of a tree can always be known on inspection. We know, however, of no possible way by which a disease arising from an aerial cause alone can be increased, when intercourse with those sick of it is increased, and diminished when such intercourse is diminished; excepting in cases where such intercourse caused greater fatigue or anxiety.

"Events of great importance to mankind frequently arise from the most trivial and ordinary causes. Thus, a beggar's blanket may depopulate a city, the mistress of the world; or, by cutting short a single life, may even affect the destiny of a whole nation. Cromwell fell a victim to marsh miasmata. The health of Napoleon was affected for years, by having seized the ramrod of a piece of artillery at the siege of Toulon, at which he had seen an artilleryman killed in the act of ramming home. Yet few great events happen without efforts being made to connect them with something deemed more worthy of themselves; the spirit of prophecy and weather-wisdom is instantly awakened, and the most ordinary circumstances are sure to be noted down and exaggerated to account for them. The following have been recorded by authors on epidemic diseases, and brought in as proof of the existence of some unusual and mysterious agency.

"Domestic animals, such as cows, horses, cats, dogs, birds in cages, in short, part of all under confinement within doors, have sometimes been observed to sicken or die during the rage of an epidemic; women with child miscarried; iron, steel, and other polished metals, corroded; leather become mouldy; clothes mothed and spoiled; beef, &c. putrefied; cheese mited; butter turned rancid; milk soured; the sky had a blood-red cast; the horizon an iron-bound appearance; the sun set every night in an unusual way; the very earth cracked or yawned, as if for its prey; the grass was alive with grasshoppers; the air was darkened with clouds of flies, and other insects; frogs croaked in the ponds, and sparrows, robins, and other small birds, disappeared from the place. Such is a specimen of the frightful catalogue in the chapter of accidents, more or less of which have been noted down, particularly by the older writers on epidemics. They have likewise been brought forward by some of the moderns of great learning and distinguished rank, in proof of what is called, for the want of a more definable name, epidemic influence: that is, something not referable to the sensible qualities of the atmosphere, or to the effects of season. That these appearances have generally been exaggerated through fear, ignorance, and superstition, can scarcely be doubted, when we remember at how late a period the laws against sorcery and witchcraft were put in force in England.* Sir Gilbert Blane found an instance on record, in the Tower of London, where a man was tried and executed for burning coals, it having been deemed, in older times, injurious to the public health. The great Van Swieten reprobated the use of soap, on similar grounds.

"Bacon, Boyle, and Locke, were, themselves, instances of superstition, even with regard to ordinary things: this fact is undeniable. Admitting, however, that every thing here enumerated had really happened in the rage of a great epidemic, they would only prove, that heat, moisture, or some of the sensible qualities of the atmosphere, had been in excess. At a time when terror reigns

* In the year 1646, two hundred persons were tried, condemned, and executed, for withcraft, at the assizes for Suffolk and Essex: see *Howell's Letters.* So late as 1699, five persons were burnt at Paisley: see *Medical Logic.*

in every bosom, self-preservation must be the predominant feeling; when relation deserts relation; when the parent is unable to administer to the wants of his offspring, or the child to aid the parent; when the dead lie in heaps unburied, and the infant perishes from hunger, at the cold breast of its mother; can we wonder if domestic animals sicken and die from the mere want of food, drink, or proper attention? Canary-birds will die, with blood at their bills, from common diseases, brought on by common causes, as want of drink.

"Unless wild animals died in as great proportion, no inferences can be drawn in proof of atmospheric causes. Pregnant women miscarry from being relaxed by a long course of hot weather; but they do so, likewise, from terror; grief for the loss of a relation; fatigue, or want of sleep in attending one. During great sickness they must be called upon to perform more than ordinary labour.

"When people wear sheets instead of shoes; when they are covered with blankets instead of clothes, and use spoons instead of knives or forks, is it surprising that leather gets mouldy, that clothes moth in the chest, and iron or steel corrodes? When out of use, and therefore too long kept, will not beef get putrid, cheese decay, butter turn rancid, milk get sour? May not all the above things happen under such circumstances in ordinary seasons? If this be allowed, it cannot be difficult to account for the rest. As flies and other insects are attracted in prodigious numbers to any spot where food abounds, so they, like other animals, multiply and increase under favorable circumstances. Fewer persons being astir to disturb them, the myriads that exist in great epidemics is readily accounted for. When frogs are not pelted by children near a large town, they, too, must breed faster and become more audacious. When accumulation of putrid smells, and accumulation of flies and other insects, attract crows, magpies, and birds of prey in greater numbers to the neighbourhood, especially at a time when every thing around is quiet and has a deserted aspect, then sparrows, robins, and other small birds, will be glad to decamp.

"In this way we explain many of the phenomena that have been noticed by authors, as proofs of the existence of a mysterious agency in epidemic diseases. At the same time, we are well aware that we shall be ranked among common observers by all those who are above making common observations. We shall be contented to remain in this humble rank; but, at the same time, we must do ourselves the justice to deny, that the observations we have made are common; for we know of no author on epidemic diseases who has given them due consideration. The phenomena above enumerated are no more entitled to the important inferences attempted to be drawn from them, than a hundred other occurrences which are every day passing around us." (P. 43.)

Dr. Aiton's observations on susceptibility are well worthy perusal: we regret that we can only extract his concluding remarks.

"These instances of exemption from contagious diseases of the most formidable kind are not singular; volumes might be filled with proof of the same kind, yet what would it serve to shew? Certainly not that plague, small-pox, and yellow fever, are not contagious diseases; but merely that no malady is alike infections at all times and under every circumstance. Mr. Hunter mentions an instance of twenty persons being bitten by a mad dog, yet only one of them took hydrophobia. Would we argue, from the circumstance of nineteen persons escaping, that the twentieth had not hydrophobia, or that this distemper was not infectious?

"The first time that yellow fever appeared in Gibraltar, only twenty-eight persons escaped it out of the whole civil population, amounting to fourteen thousand. Would we argue from the fact, that every individual who had been attacked with the disease the first time escaped it on another visit, (viz. 3,800 out of 7,370,) that yellow fever is not contagious? Yet it is astonishing to reflect how much this negative evidence has influenced the minds of medical men. The most trifling and casual change in the feature of a disease, the simultaneous or even previous occurrence of other diseases in men or in cattle, the slightest deviation in the rise and progress of the contagion at one time from another, its observing laws somewhat different from those which govern other contagions, or the most ordinary appearances of weather, are circumstances which have all been eagerly laid hold of, and greatly exaggerated, with a view to assist and prop up this lame negative evidence. The occurrence of any two of the above circumstances, during an epidemic, is deemed quite sufficient to stamp its character, and overturn the experience of ages; whilst differences in point of exposure to contagion, and the susceptibility of those exposed, are generally left out of view. A smaller proportion of persons exposed to the infection will have hydrophobia, than, perhaps, any other contagion, and the disease will be much longer of manifesting itself in those who take it. Some authors compute that only one in sixteen, bitten by a rabid animal, will take the disease; and most agree that it may remain dormant in the system for years before infection is certain. The important circumstance of the bite, however, is not soon forgotten; hence no one doubts that canine madness is infectious. In cases of infection from other diseases, they have nothing to speak home to the feelings; they, therefore, proceed on the erroneous principle, that, because a man does not know when and where he has been bitten, he is not bitten at all." (P. 69.)

Of the difficulty of tracing the line of *communication* of diseases, which are nevertheless communicated from one person to another, and not unfrequently through the medium of a third, who, although the carrier, may, from insusceptibility, remain unaffected, the following, among other instances, is given:

"Another cause of the difficulty of tracing contagion is the non-susceptibility of certain individuals to its influence. If some persons receive or even wear infected clothes without experiencing any bad effects therefrom, a link in the chain of discovery is thus lost, and often leads to very erroneous conclusions concerning them.

"A very interesting case of this kind is mentioned by Tully, which happened during the plague of Corfu. The complete success of the attacks made on plague by British bayonets, directed by the indefatigable generalship of His Excellency the late Sir Thomas Maitland, will long be remembered. This distinguished individual, alike regardless of all opinion, and deaf to the scientific objections of medical men, proceeded at once to suppress pestilence, in the same way he did to bear down piracy, and in both instances the strong arm of power was equally successful and conspicuous. At this time, two Greek lovers who were betrothed to each other, happened unfortunately to be separated by one of the lines of circumvallation, part of which was bounded on one side by a river. The swain, on this occasion, chanced to belong to the proscribed or infected district; the maiden, a beautiful Greek girl, to the healthy, and the house in which she lived along with her mother, was situated close to the bank of the river. For a time the two lovers could have no communication, except now and then holding a stolen conversation from the opposite banks of the stream, perhaps to inquire after each other's health. So long as the ardour of their love was thus restrained, all was well; but the young Greek on one occasion threw a purse containing some money over the river, which was immediately picked up by the girl's mother, who, after keeping it some time, gave it to her daughter. The consequence of this act of kindness was the death of the latter: she put the purse, on receiving it, into her bosom, and soon afterwards died of plague. The mother, who was the first to receive the purse, remained unhurt, although she kept it in her possession for some time." (P. 197.)

On "*the evidence of contagion*" our author reasons well: the section on this subject we recommend to the attentive perusal of our readers. "Instead of drawing important conclusions from negative proof, or by making partial appearances, the criteria of general judgment,

"Would it not (as he observes) be more advisable to investigate contagion by the rule we judge of other matters, the existence of which is not susceptible of direct proof, that is, by their effects? For instance, the question may be asked, is the sun a sourceof caloric or heat? this question cannot be decided by direct demonstration. Caloric itself is invisible, untangible, inponderable, and unappreciable, except by its effects. Nor have we direct proof of its being derived from the sun. When that luminary arises in the eastern horizon, the well-known effects of caloric are instantly perceived; as it approaches, the heat increases in proportion, and it declines again as the sun goes down or retires. When objects intervene and prevent the free passage of the calorific rays of the sun, the effects of heat are not felt beyond the obstructing media. If the rays be concentrated by a glass which allows of their being freely transmitted, the heat is prodigiously increased in power. This is deemed evidence sufficient that the sun is a source of heat.

"Is measles a contagious disease? This is not susceptible of direct proof. No one ever saw the contagion of measles; no one ever weighed, or to his knowledge touched, it. Like caloric, it is only appreciable by its effects; for we have no direct proof of contagion being derived from the body of a person labouring under measles. When such a person approaches near to one in health, the effects of contagion are instantly perceived on him by a sick smell. As both persons approach each other, the effects are increased in proportion. They are diminished as they retire from one another. If any body be placed between, so as to obstruct the passage of the emanations from their source, the effects are not felt beyond the obstructing media. But these emanations can be collected and concentrated, so as to increase their effects to a prodigious degree. The proof then that contagion is derived from measles is just as conclusive as that heat is derived from the sun. Can the presence or quantity of caloric in a body be always perceived by its usual effects? No; caloric may be present in a body without its effects being perceptible. The same thing takes place in contagion. Are all persons alike sensible to caloric? No; some are more susceptible than others: so it happens in contagion. Can the latent caloric existing in a body be called into action by a change of circumstances? Yes; so can latent contagion in the animal system be excited. Are all inanimate bodies alike capable of conducting or transmitting caloric? No; some conduct and preserve it much better than others: this too with contagion. Does caloric spread from any given point in all directions with the same facility? No; it spreads in certain directions, and with greater or less rapidity in proportion to the quantity and quality of the combustible materials that happen to be present; so does contagion. If caloric has destroyed the inflammable materials which existed in a body by passing through it, is that body again capable of combustion? No; neither is a human body capable of being again infected with the same species of contagion. Has the atmosphere much influence in regulating or affecting caloric? Certainly; so the matter stands with contagion. Thus, then, the method of judging by induction and by analogy, may often lead us to the most correct and certain conclusions. What would be the results were we to adopt the method by allowing negative to outweigh positive evidence. Crowding, filth, nastiness, intercourse and communication with those labouring under contagious diseases, do not always engender or propagate contagion. We have given some satisfactory reasons why they do not." (P. 118.)

To those who are not conversant with the doctrine of chances, and contend that diseases are not communicable because they are not always communicated, we recommend the following to be reflected on.

"Much has been said about this want of what is called direct proof, and many very erroneous conclusions have been drawn from it; but, if we apply the doctrine of chances to the facts we have brought forward, what would be the result? I proposed, says Dr. Stokes, the following problems to a friend, particularly acquainted with this species of computation. 1st. An epidemic prevails so severe, that one of seven sickens: a family of twelve is selected in a particular district before the epidemic has visited it: what is the chance that eleven out of this family should take the disease, supposing the sickness of one of the family does not promote the sickness of another, and supposing the family not unusually liable to disease? The answer is, that the probability against the event is nearly 189,600,000 to 1, if the population amount to 7,000. 2d. The same conditions being assumed, what is the chance that, in any family of twelve, within the district, eleven will sicken? Answer; it is above 300,000 to 1, that no family of twelve will have eleven sick. —Vide *Stokes on Contagion*, pp. 23-24. Let us apply the same principles to the epidemic cholera now prevailing in Sunderland, where six in one family were attacked, five of whom died. Let us suppose that Sunderland contains seven thousand inhabitants, and that one thousand out of the seven had been attacked with the disease, the chance that six, which in the Sunderland case we believe was the whole number the family contained, sicken in one family, is 117,649 to 1 against the event. But the population of Sunderland being more than twice this number, and those actually affected only 400, the probabilities against it were infinitely greater." (P. 210.)

"Although the bad effects of a wrong opinion of contagion are seldom, if ever, confined to those who embrace it, but prove equally destructive to others; yet we know no subject on which speculation is oftener indulged or exercised, and certainly none in which mankind have profited so little by past experience. Every one who has heard the terms contagion and infection, and believes he understands their true signification, seems to think himself justified in promulgating his opinion. The tyro in medicine who has seen a few cases of cow-pox or measles, the quack who owes his reputation to the ignorance of his patients, and the parent who has observed disease chiefly within the circle of his own family, frequently boast of having made up their minds on the subject; and it will generally be observed, that each individual becomes stubborn or otherwise in proportion to his ignorance. We cannot, therefore, too strongly warn the public of the danger of listening to the crude and flimsy effusions of the newspaper press, on the subject of cholera and contagion in general. Many of these productions have evidently been written by men of some talent, but who think it a mark of greater wisdom to dissent from the opinions of the better-informed and more experienced. According to them, age is imbecility, caution is cowardice, wisdom is folly, and knowledge any thing but power.

"Two years ago we gave it as our opinion, that the Asiatic Cholera is contagious, and that it would ultimately reach this country: we see no reason to alter this opinion. Much has been said about contingent contagion: what contagion does not depend upon contingencies? Throughout the whole of this dissertation, therefore, it has been our particular study to point out the necessity of attending to these very contingencies or concurrences of circumstances, before we can account either for the generation of a contagion or for its after-spreading. Cholera is an exotic plant, and may not be able to spread out its roots and branches so easily in Britain as it does on its native soil. That imperfect and spurious forms of the disease may be more frequent with us, and that such forms of the malady are not contagious, are things not to be wondered at by any one who carefully peruses the foregoing sheets; but, had we no other facts to prove its contagion than those furnished by the history of its ravages in Sunderland, these would warrant the wise precautionary measures adopted by government.

"An agent floating in the atmosphere of a place is just as likely to affect one individual living in it as another, supposing that all are equally susceptible of its influence, for we know of no way by which that atmosphere can be breathed by one person and avoided by another. Let us suppose that the town of Sunderland and its immediate neighbourhood contains 15,000 inhabitants, which we believe is nearly its population, and that five hundred persons have been attacked with cholera, then one person in thirty would be the proportion of sick on the atmospheric theory; but if one individual out of a family containing six persons was seized, then it would have five times its proportion of sick; so that even in this case the balance would be in favor of contagion. What then must be our conclusions, if six, the whole number the family contained, be attacked with the disease, and five out of the six die, the chance against such an event is some hundreds of thousands to one.

"Again, the cholera of Sunderland has already attacked more than four hundred persons, including the mild cases, and it has proved fatal to more than one in three and a half. Yet we are told by some that it is only a British disease, and one derived from the atmosphere. We have before given the annual number of deaths in London from bowel-complaints, (which includes cholic, flux, gripes, &c.) taken from an average of ten years, at the beginning, middle, and end of the eighteenth century, when the whole deaths were about 21,000. By this document, viz. the bills of mortality, it appears that 1,100 died of these complaints in one year at the beginning, 135 at the middle, and only twenty at the end of that century; so that out of a population of about 600,000, only twenty persons, or one in thirty thousand, die of every thing in the shape of bowel-complaint in a whole year in London; whereas, in Sunderland, out of a population of 15,000, one hundred persons die, or 1 in 150, of one single form of disease in a few weeks, and that too in a mild season, and in an age when the mode of living is so much improved. Query: If a British disease kills only twenty persons out of six hundred thousand in one year, what is the probability that a malady which kills five out of six persons in a family in a few weeks, is also a British disease?" (P. 215.)

We have read these dissertations, and we are sure they will be read by others, with much interest and attention: we trust they will effect that good which they are so well calculated to accomplish; and although, from the subsidence here of that disease which so fearfully threatened us, there may be less present need of the exhortation with which Dr. Aiton closes the volume than there was at the time it was written, still as the danger is not gone, although it is retiring, and as, even if it had departed, they are applicable to other calamities which are constantly with us, we cannot refrain from letting the author conclude the subject in his own words.

"Let us unite, therefore, in endeavouring to avert, or at least to lessen, the evil of this awful visitation. Let the ignorant and prejudiced be informed, the selfish and interested abate their desire of gain; let the pedant pause before he promulgates his opinions, the libertine cease to boast of his freedom; let the politician dismiss from his heart the rancour of party spirit, the dogmatist learn from past experience; let the indolent rouse from their slumbers, and the rash become acquainted with danger; let all these read the history of cholera.

"This scourge, of supposed atmospheric origin, has travelled in a slow and gradual manner over a great portion of the civilized world, keeping pace not with the winds, but making its journeys at the rate of travelling in the countries it visits. No climate, season, or soil, can retard its progress: it rages in hot, in cold, and in temperate regions; in high winds and in calm weather, in great droughts and in torrents of rain, in mountainous districts and in level plains, in countries well cultivated, and in those in a barren or desert state; on places rich in soil, and places having no soil; it has travelled over seas and over rivers, in the face of a gale of wind or with the wind; it is regardless of the laws which regulate caloric, magnetism, or the electric fluid: in short, it mocks at repulsion, holds out its finger at chemical attraction, and stalks in terrible array over every species of matter; and what is the reason of all this? The answer cannot be mistaken; it is nursed in the lap of society, and conveyed from place to place by intercourse and communication." (P. 220.)

*Lithotrity and Lithotomy Compared; being an Analytical Examination of the present Methods of Treating Stone in the Bladder, with Suggestions for rendering Lithotrity applicable to the Disease in all its Stages and Varieties, and Remarks on the General Treatment of Gravel and Stone.* By THOMAS KING, M.D., M.R.C.S., Surgeon to his Excellency the French Ambassador, &c.—8vo. pp. 320; three Plates. Longman and Co. London.

WE are in general intolerant of all those works which, while professing to discuss some practical point, either in anatomy, surgery, or any of the collateral sciences, such as the title-page of this book announces, (points which should be submitted to the consideration, and decided by the voices of veteran practitioners alone,) nevertheless devote, as not unfrequently is the case, a third, or even more than half of a small volume to a superficial account of matters which are known to every tyro, and are much better stated in almost every primer. Therefore, when we opened this work, and found that "Lithotrity and Lithotomy compared" were to be ushered in, or rather kept back, by a preliminary treatise, or rather series of dissertations on the anatomy of the pelvic viscera, such as, 1, a "sketch of the urinary apparatus;" 2, "description of the bladder in the adult male subject;" 3, "description of the urethra;" 4, "organization of the bladder and urethra," &c., we will confess that we made up our minds, with all due patience and submission, to be tortured with the thousand and first repetition of the common-place anatomical truisms which authors, who want to make a large book out of a little matter, so recklessly appropriate, and so unmercifully inflict on their readers and reviewers: but for once we were wrong in our anticipations, and we therefore the more readily grant that we were agreeably disappointed with the perusal of Mr. KING's introductory chapters; for, with a very commendable brevity, he has condensed the five anatomical essays into less than ninety-five pages, and, even had he thought proper to extend them a little further, we should not (after reading them) have complained; for we think his description of parts that are very important, and which we have thought in general too little dwelt on in elementary books, the most satisfactory and perspicuous that we have seen. There are two or three points likewise which are new, or at least which have not, on the whole, been heretofore so emphatically insisted on, but which, nevertheless, we are of opinion that the author has rather too formally announced: and the one that he more especially dwells on certainly is not entirely new to us: we well recollect Mr. CARPUE, in a public lecture he gave many years ago on the high operation for the stone, pointing out the very little portion of the bladder which remained uncovered by peritoneum, even when that viscus is fully distended; as the distention raises the fundus, which, with its peritoneal covering, is felt through the abdominal parietes; and he most particularly impressed it on the attention of his hearers that, even when distended to the utmost, so as to rise above the pubis, its attachment to the symphysis would prevent it "passing to a *considerable extent* between the peritoneum and the abdominal muscles, or rather between it and the fascia transversalis." Indeed, if our memory serves us faithfully, this is most clearly shewn in a plate accompanying that gentleman's work on Supra-pubic Lithotomy, published in 1819; a book which we presume Mr. King has not seen, otherwise he would have noticed it in his own account of the high operation. Such being the state of the case, we are of opinion that the following passages will require considerable modification:

"The anterior region of the bladder corresponds to the symphysis pubis; to the pubic ligament, from which it is separated by its own anterior ligament and cellular tissue; and, in a small extent, to the triangular ligament of the urethra. Above the symphysis, this region corresponds, opposite the linea alba, to the fascia transversalis of Sir A. Cooper: but whenever the bladder rises fairly an inch and a half above the pubis, it is in contact with the peritoneum lining the wall of the abdomen, in addition to its own peritoneal covering. In other words, the shining surface of the peritoneal covering of the bladder is in contact with the same surface of the peritoneum lining the muscles of the abdomen; so that an instrument, to penetrate this part of the organ, must traverse the peritoneum twice.

"I am induced to lay claim to the discovery of this fact; for all the authors I have read, state, that when the bladder is distended so as to rise above the pubis, it passes to a considerable extent between the peritoneum and the abdominal muscles, or rather between it, and the fascia transversalis. I was led to this discovery, from having seen the peritoneum wounded in the high operation for stone, by the best operators; which I could not explain, till I observed, on investigating the subject, that, when the bladder is distended by insufflation, it rises in the proper cavity of the peritoneum. I do not pretend that a small part of the bladder, thus distended, may not be uncovered by the peritoneum above the pubis; but I positively assert, that this organ, (and in old persons more especially,) expands in the abdomen, in some such manner as the uterus does in gestation, by a gradual yielding of its peritoneal as well as of its other coats; and not by detaching the peritoneum, as it has been hitherto supposed, from the abdominal parietes. It is remarkable that an acquaintance with the nature of serous membranes did not lead *a priori* to a knowledge of this fact. Why should not the serous membrane of the bladder yield as much as its mucous and muscular coats; when it is well known, that the susceptibility to yield to distention is one of the characteristic properties of serous membranes? Indeed, they yield more promptly than other membranes, as we see in hydarthrus, hernia, ascites, and in a multitude of other circumstances.

"I need not insist more upon the importance of this anatomical disposition, particularly as it will be necessary to return to the subject hereafter; but it explains at once the error committed by lithotomists, who, in laying open the bladder above the pubes, to their great consternation and surprise, have found their fingers or instruments in the cavity of the abdomen." (P. 30.)

We doubt not that Mr. King made the discovery that here he claims, and that he believes himself to be the first anatomist who has made the observation; but we, who saw the whole, or nearly the whole, of the cases which were operated on above the pubis by Sir Everard Home, Mr. Ewbank, and Mr. Rose, at St. George's Hospital, shortly after Mr. Carpue brought Souberbielle's instruments to England,—we who, feeling much interest in the subject, attended many of the demonstrations and experiments which then were being made on the dead subject, well know that this point did not escape notice; and, indeed, some of Souberbielle's instruments were stated to be expressly designed to open the bladder through the very small space which rises uncovered by the peritoneum above the pubis, and the liability to infiltration of urine was endeavoured to be lessened by making the counter-opening which he recommended in the perineum, and to the omission of which in the English operations its ill success in this country was by many attributed.

In instituting a comparison between two series of operations, it, of course, becomes necessary to give a general description of both; nevertheless, although we admit the author's obligation not to neglect the account of the lateral operation, we do not admit our obligation to make any very copious extracts from the descriptive portions of this chapter: we shall, therefore, confine our quotations to the summary of the effects of violence in extracting large calculi by the peritoneum, which are correctly and feelingly detailed, and we think are worthy of serious consideration; for, although they have frequently before been stated, they never can be too frequently repeated.

"Of the patients who submit to the lateral operation, one in seven or eight dies; and, in almost all those cases which have a fatal issue, death is produced either by the force used to extract the calculus or by too extensive an incision in the prostate. If the incision is small in comparison with the stone, death will follow from the violence done to the bladder and surrounding parts, in the extraction; and if the incision is made sufficiently extensive to admit of the fair extraction of a stone one inch and a half in each of its two lesser diameters, death will follow from infiltration of urine.

"In criticising the lateral operation, the first thing to be attended to is, then, the volume of the stone; success or failure depends upon it. If the foreign body never exceeded three inches in its lesser circumference, so that the incision in the prostate might be limited to three quarters of an inch or a few lines more, the operation, when well performed, would seldom or never be followed by fatal consequences. When it measures four inches and a half in its lesser circumference, or that the sum of its two lesser diameters amounts to three inches, the patient may recover, but the chances are very much against him; and when it exceeds this volume, death is almost sure to be the result of the operation.

"The surgeon is in this dilemma; he must either use force, or make a long incision: the former lacerates the prostate and cellular tissue, bruises the bladder and stretches its membranes, and shocks the nervous system; the latter prepares the way for infiltration of urine: both are fatal nearly to the same degree.

"Let us proceed to the proof of what has been advanced. We have seen that the largest transverse diameter of the pelvic outlet, taken from the bare bones, is only three inches, and that opposite the prostate it is only one inch and three quarters; it should be recollected too, that as the incision is made only on one side, the whole of this space is far from being available. When the stone is drawn, as it should be, round the back part of the incision, and then in a direction nearly parallel to the superior aperture of the pelvis towards the rectum, the parts pressed between it and the inferior margin of this cavity will certainly yield considerably; but more than an inch and a half will seldom be obtained by moderate pressure.

"But the great obstacle to the extraction of a stone measuring an inch and a half in its lesser diameters, is the situation of the prostate. The calculus must pass between the inner surface of the rami of the ossa pubis, and the posterior boundary of the incision in this gland; and, as the distance between these, when the incision in the prostate is not dangerously extensive, is only an inch and a quarter, it necessarily presses the anterior wall of the bladder against the bones, on the one hand, and tends to tear the back part of the prostate on the other. As the postate is firmly fixed, it cannot be drawn backwards, or pushed farther from the bones towards the cavity of the pelvis, without the ligaments yield or break: yield they may, perhaps, a quarter of an inch without laceration, and then a stone of the above size may be extracted, possibly without mortal injury.

"But, when the foreign body is larger, something must give way: the bones cannot yield, and if the calculus be not crushed, the prostate and bladder must be torn; and the effects of this laceration is almost certain death.

"If we have described the parts correctly, the inference that laceration must occur either in the prostate and bladder, or in the tissues by which they are fixed, cannot be disputed.

"The next question will be, ought not this to be prevented by an extensive incision? But, before entering upon it, we have to prove the truth of the last conclusion, that the effect of laceration is death.

"The experienced surgeon will admit, that in one half the number of cases which terminate fatally, death is the result of force used to extract the stone; and whoever peruses attentively the history of such cases detailed with the post mortem examinations, will come to the same conclusion. But, setting aside, for the present, the positive evidence afforded by these cases with which the records of surgery teem, and which are familiar to the memory of almost every practitioner, let us look at the natural effect of those injuries, which are inseparable from the employment of force in the extraction of calculi.

"In the first place, when the prostate and bladder are torn or much contused, death will often result from the immediate shock of the injury. Secondly, if the fascia forming the capsule of the prostate, or that constituting the ligaments of the bladder, or even the neighbouring cellular tissue, be torn or severely bruised, death will ensue from suppuration and sloughing occurring either in the cellular tissue of the pelvis, or in some tissue or organ more or less distant from it. Thirdly, the same result will follow from the infiltration of urine, which is so frequently produced by the above injuries.

"It is universally admitted that the contusion of any internal organ is almost necessarily mortal. A patient rarely survives contusion of the brain, lungs, heart, liver, spleen, kidneys, stomach, or intestinal canal; if the arachnoid membrane, the pleura, pericardium, or peritoneum be stretched or torn to any extent, death will soon follow; indeed, if the injury be only very slight, it will be almost always succeeded by inflammation of a kind which rarely admits of recovery. How then can it be supposed that similar injuries of the bladder, the bladder composed of peritoneum, mucous and muscular membrane, the bladder with its abundant nerves and vessels, and its venous plexus; the bladder containing urine, and surrounded on all sides by aponeurotic structures; the bladder so intimately connected with the genital organs, are exempt from a similar result?

"If it go hard with life when a large joint is violently distended, a muscle torn, or a nerve lacerated, what are we to apprehend when such injuries are inflicted upon the urinary reservoir?

"The use of force to extract a stone is so surely followed by death, that I shudder to think how often surgeons have recourse to it. It has so generally occurred to me to fortell the issue of a case simply by the degree of force employed, that, if I witness it now, I do not hesitate in indicating to those near me when the operator has arrived at the point beyond which recovery is impossible. I shall shew presently, that a large opening in the prostate, made scientifically with the knife, is too dangerous a lesion to be allowed much longer to belong to surgery; but dangerous as it is, one would almost call it a safe and simple wound, when compared to that inflicted by a surgeon, who, placing his foot against the table, employs all the strength he possesses to stretch and tear the bladder, lacerate its connecting tissues, bruise the surrounding parts, and shake, mortally shake, the whole nervous system! This anti-physiological process, this absurd and horrid practice, is so cruel, so fatal, and yet so common, that there is nothing in which, as a surgeon, a man might more justly pride himself than having contributed to abolish it. But let us pursue the investigation.

"When the bladder is full, a violent contusion on the hypogastric region, such as we sometimes see from the kick of a horse, is frequently followed by suppuration of the cellular tissue in the vicinity of the organ, and in this event always by death. In extracting a stone with force through an opening in the perineal portion of the bladder, we produce the same kind of injury; there is, however, this great difference, that the parts interested are much more vascular, nervous, and delicate, in the latter than in the former case.

"I could refer to individual operations of lithotomy, where patients have expired on the table or a few hours afterwards, from the shock communicated to the nervous system by the forcible extraction of the stone, or to those less immediately but as certainly fatal, where the patient has lived weeks without ever being able to rally; but they are too notorious, too much every-day occurrences, to require detailing in this place. If it ever occurred to the operator, during the athletic efforts made to extract the calculus, to ask himself what he was pulling at, would not the answer which anatomy suggests compel him to desist? he is not merely dragging out a stone, and pulling at the bladder, its delicate and tender membranes, its vessels and important connexions; but at its nerves, and, by means of these, at the hypogastric plexus, the great sympathetic, and spinal marrow! This is the simple physiological explanation of what follows the operation, or the plain analysis of what is called the shock communicated to the nervous system by wrenching a stone with force from the bladder." (P. 104.)

After some good practical remarks on infiltration of urine, which so commonly occurs after violent efforts to extract a calculus, whereby the prostate and adjacent tissues are lacerated, and the enlargement of the wound by the knife to avoid laceration, our author continues:

"It would be difficult to say geometrically how long the incision may be made without danger; and I certainly have not the presumption to draw precisely the line, on one side of which is safety, and on the other death; but I am convinced, from meditation on the anatomical facts connected with this subject, and from the painful experience of seeing many die, that the danger of a wound in the prostate is in the direct ratio of its extent, and that an incision necessary for the extraction of a stone measuring an inch and a half in its two lesser diameters puts life in imminent peril.

"Of the two evils, the use of force, or a long incision, there can be no doubt which is the minor; since the latter subjects the patient to only one serious danger, whilst by the former he is exposed to many fatal consequences.

"My opinion is, that surgery should claim as an axiom, that a large incision is always preferable to force sufficient to contuse and lacerate the prostatic, or any other part of the bladder, or the fibrous tissues surrounding it; but surely the lesser evil will be considered too serious to be tolerated, when it is recollected that nearly half the patients who die succumb to the infiltration of urine it occasions.

"Nothing can exceed the dread which lithotomists have of a large incision; their instruments are almost invariably constructed so as to prevent it: yet what can be done? This is the question always put to himself by a good operator, upon discovering that he has to deal with a large stone; a question we hope to be able, as it is a part of the design of this work, to answer. I have frequently been a witness, and I may say a sympathising witness, of the terror and extreme embarrassment felt by the surgeon at the moment of this discovery: if he thought of enlarging the wound, the certainty of exposing his patient to infiltration of urine would flash across his mind; and, on the other hand, his knowledge of the inevitable consequences of lacerating and bruising the parts, would incapacitate him for having recourse to force. Many, I admit, do use force, in the hope of dilating the wound, as I have already observed; but this is not possible to any extent.

"By these facts we may judge, then, how reluctantly operators adopt a long incision: and if laceration of one of the most delicate, most sensible, and most important of the internal organs be preferred by some operators to a large opening made with the knife, and that both means are had recourse to only as their desperate *pis aller*, surely it is high time to think of treating stone in the bladder by some more rational process than lithotomy as it now stands." (P. 119.)

The chapters treating of the Recto-vesical and Supra-pubic Operations we shall pass without further comment, as we do not think they will ever be put in competition with lithotrity, improved and improving as now it is.

The preliminary precautions, and the successive steps of the operation of lithotrity, have already been detailed in our pages, when giving an account of CIVIALE's and HEURTELOUP's claims and modifications; therefore, on them we shall not now dilate, and shall only extract the description of the Baron's *percussor*, to which we formerly alluded.

"Some attempts have been lately made by Baron Heurteloup to destroy calculi by percussion alone. The instrument which he has invented, and used with some success, resembles, when closed, a large, solid, curved sound: it is composed of an internal or superior, and an external or inferior piece of steel; and the former is included and made to slide, to and fro, in a deep grove of the latter; so that their curved portions, which are beset with teeth, strike one against the other, and abutting, constitute a kind of vice for seizing and acting upon the stone. At the extra-vesical extremity of the instrument the internal piece projects, and is so fashioned that it may be struck with a hammer, the weight of which is proportionate to the force that may be employed with safety; it has, also, a stop to prevent its being driven with too much violence against the external one, which is made with a square head to fix immoveably in the holdfast of the bed. It is proposed to use this instrument in the following manner: The operator passes it down the urethra into the bladder closed, and, after finding the calculus, opens it by drawing back the internal piece. He then endeavours to engage the foreign body between the blades or curved parts of the instrument, and having succeeded, he is to fix the square head of the external piece firmly to the bed. At this time, the bladder, being filled with water, is protected from the shock that would otherwise result from the process. The surgeon now strikes smartly, with a hammer, the cap-shaped, projecting end of the internal piece, which thus becomes a percussor, until the stone yields." (P. 194.)

Several interesting cases are detailed, in which the patients have been treated in various ways; but these we pass, to make room for the following summary:

"The great character by which Lithotrity is distinguished from, and elevated above Lithotomy, is, that it accords far better with the recurrent nature of calculous disorders. When once the kidneys have taken upon themselves to secrete gravel, it is difficult to put a lasting stop to the habit. Whatever means are adopted, there always remains a certain predisposition to resume an action that has before existed, and the slightest causes may occasion a relapse. If, then, the disease is liable to return, and to return frequently, it requires a remedy that can be repeated without the risk of life: such is not lithotomy; but approaching to such, appears to be lithotrity. When a patient is cut for stone, the operation puts life in danger, yet it is no protection against a return of the disease; and, as often as it is repeated, so often does the patient risk his life. Lithotrity, on the contrary, does not endanger life: it can be repeated with safety, and applied with effect, at the first moment of a relapse; it has also the great advantage, that the patient can contemplate it without the dread which lithotomy creates, not only in him, but even in the operator himself.

"It was a happy thought, that of reducing a stone in the bladder to a state which admits of its passing away with the urine. While in the form of gravel, urinary concretions are expelled by a natural process, in fact, by the exercise of the organs in which they are lodged; nature has this resource, until they become too voluminous to escape through the urethra: what, then, can be more rational than to restore to her this power, in making them re-assume the form of gravel, by acting upon the morbid product, rather than by maiming a healthy organ? We do not pretend, that, in the present state of lithotrity, the morbid production can be got rid of without great pain, and some injury to the organ containing it; but this is owing to the deficiency of our instruments, and not to any defect in the plan. Few operations, indeed, are so well founded as lithotrity; and, when once we possess instruments a little more perfect than those now in use, stone in the bladder will not be deemed a more serious disease than gravel." (P. 231.)

"It should not be forgotten, that a cure is not very promptly obtained by lithotomy: after cutting, the patient remains in a very critical state till the wound is healed, and a long convalescence is always the result of this severe operation.

"With regard to a relapse of the disease, we admit that it may be more frequent after lithotrity than after lithotomy: the nature of the disease, however, is such, that the latter operation is no protection against it. Indeed, there is but little trouble taken, after cutting, to clear the bladder of any fragments which may have been broken off with the forceps; the surgeon is anxious to get his patient to bed as soon as possible, knowing that the operation itself is quite enough for life to struggle with. On this account, perhaps, the formation of a new stone is very nearly as common as after lithotrity. Relapse after any surgical operation is not only dreadful to the patient, but extremely mortifying to the surgeon, whom it disheartens, by impressing him with, perhaps, an inordinate conviction of the want of permanent efficacy in our remedial resources; but, if there be a cure where relapse is less unfortunate than in others, it is surely that in which the remedy is always at hand, and can always be applied without risk of life. It is impossible to repeat too often, that the grand and characteristic advantage of lithotrity is its appropriation to the nature of calculous affections, from which no period of life is exempt, and which, depending in a very great degree upon idiosyncrasy, may be liable to recur every three months. I well remember the case of a youth who was cut for stone, at the Hôtel Dieu, in whose bladder upwards of two hundred small calculi were found. He died from the severity of the operation. Now, his life would have been saved with ease, had lithotrity (then unknown) been applied as each calculus descended into the bladder from the kidneys. I verily believe, that, excepting children, a very large portion of those operated upon by lithotomy have been afflicted with a relapse. Several of such cases have come under my own immediate observation; and, on one patient, I saw the operation performed for the third time. Before the invention of lithotrity, no one at all acquainted with the subject, not even the most courageous man that ever existed, could contemplate the recurrence of this terrific disease, without the most awful apprehensions; but, now, I see patients calm, and hear them exclaim, in reference to fresh symptoms, 'Oh! it will only be a little more grinding."' (P. 241.)

"There are, doubtless, some cases where the stone is too large to be grasped with the lithotritic forceps, and others, where it is so hard as to resist the action of any instrument that can be passed down the urethra. But the number of the former will diminish every year, as the easy means we now possess for destroying a small calculus come to be generally employed. The operation of lithotomy was considered so serious, and even the name of it was so frightful to patients, that cradling themselves in the hope that their disease might be some other than stone, they did not dare to apply for medical advice, which might convert their worst fears into the much dreaded reality, until the severity of the symptoms made life almost insupportable. Were the calculus large or small, there was no other alternative than submitting to a wound in the bladder. It is on this account, that surgeons have so frequently had the misfortune to find a large stone. But, now, patients will be anxious to ascertain, at the first moment they experience any thing like symptoms of the disease, whether it exist or not, knowing that the sooner its presence is revealed, the more prompt and less inconvenient the cure; and, that when attacked at the onset, it is to be vanquished without requiring other sacrifice of them than submission to pain, which, although severe, is of but short duration." (P. 246.)

"After this inquiry into the principal circumstances connected with the treatment of stone, by lithotomy and lithotrity; in which we have attempted to place side by side their respective dangers and advantages, by an appeal to facts, and thus to establish the degree of estimation in which each ought to be holden in practice, our conviction is that, wherever lithotrity can be employed, lithotomy should never be thought of.

"Whether we look at the structure of the parts concerned, at the nature of the disease, or at the results furnished by experience, we are led to the same conclusion. Every thing conspires to establish the superiority of lithotrity, and to place it at an almost immeasurable distance above lithotomy. A wound in the bladder, of itself, endangers life, more or less, even when complicated by the serious accidents we have had occasion to notice, and every one of which may separately cause death. On the other hand, the objections that can be made to lithotrity, however numerous, are but as dust in the balance, when weighed with those which belong to the other plan; and, were we to attempt to express, in a few words, the verdict imperatively called for, by the testimony of reason and experience, that verdict would be, the abolition of lithotomy." P. 249.)

The "Proposals for the Treatment of Calculi of great magnitude and density" exhibit that fertility of resource which should always characterize the philosophic surgeon: still, although we admit their ingenuity, we cannot say much in their recommendation, especially as we trust that with lithotrity, even in its present state, and supposing it to admit no further improvements, which is highly improbable, stones of the magnitude to require such a fearful appareil as the supra and infra pubic forceps, are not likely to be suffered to occur. Still, for the cure of such cases as are now so far advanced that the lithotritic instruments cannot, when passed through the urethra, grasp the stone, "perineal lithotrity," as here recommended, will undoubtedly merit the surgeon's attention; for the details of which we must, however, refer the reader to our author's volume, which is really a very creditable performance, calculated to be of service to the profession, and to convey much consolation to those who are afflicted with that heretofore dire disease, stone in the urinary bladder.

### COLLECTANEA.

